# Forming Limit Analysis of Thin-Walled Extruded Aluminum Alloy Tubes under Nonlinear Loading Paths Using an Improved M-K Model

**DOI:** 10.3390/ma16041647

**Published:** 2023-02-16

**Authors:** Haihui Zhu, Yanli Lin, Kelin Chen, Zhubin He

**Affiliations:** 1 School of Mechanical Engineering, Dalian University of Technology, Dalian 116024, China; 2 Department of Integrated Systems Engineering, The Ohio State University, Columbus, OH 43210, USA

**Keywords:** forming limit, nonlinear loading path, M-K+DF2012 model, ductile fracture criterion, extruded aluminum alloy tube

## Abstract

To meet the requirement of lighter weight and better performance in tube hydroforming, one of the most important tasks is to accurately predict the forming limit of thin-walled tubes under nonlinear loading paths. This work established the M-K+DF2012 model, a combination of the M-K model and the DF2012 ductile fracture criterion, for the forming limit prediction of thin-walled tubes under nonlinear loading paths. In this model, the failure of the groove is determined by the DF2012 criterion, and the corresponding strains in the uniform region are the limit strains. The limit strains of an AA6061 aluminum alloy tube under a set of linear loading paths and two typical nonlinear loading paths were tested. Parameter values of the M-K+DF2012 model for the tube were determined based on the experimental limit strains under linear loading paths, and the limit strains under the two nonlinear loading paths were predicted. Then the strain-based forming limit diagram (*ε*-FLD) and the polar effective plastic strain FLD (PEPS-FLD) of the tube under different pre-strains were predicted and discussed. The results show that the limit strains of the tube are obviously path-dependent, and the M-K+DF2012 model can reasonably capture the limit strains of the tube under both linear and nonlinear loading paths. The predicted *ε*-FLD shows a strong dependence on the pre-strain, while the predicted PEPS-FLD is weakly strain path-dependent and almost path-independent on the right-hand side for the AA6061 tube.

## 1. Introduction

Tube hydroforming is one of the advanced manufacturing technologies to form lightweight tubular parts and meet the lightweight requirement in industries such as the aircraft, aerospace, and automobile fields [1,2,3]. Tube hydroforming processes often involve complex contact conditions and combinations of internal pressure and axial force [4], and even include several pre-deformation steps before the final bulging. Therefore, the deformation history during tube hydroforming is complex, and the strain path is nonlinear even in a simple tube hydro-bulging test with no axial feed [5,6].

As a type of lightweight material, aluminum alloy has been widely used in key structural parts and is being increasingly applied, especially in the automobile industry. However, the ductility of the aluminum alloy at room temperature is relatively low, and the most common challenge in the hydroforming of an aluminum alloy tube is fracture, which limits the development of the product for lighter weight and better performance. Therefore, it is important to accurately predict the forming limit of aluminum alloy tubes under complex loading conditions and avoid fracture by optimizing the loading path [7,8]. Considering that the occurrence of necking before fracture usually reflects the failure of the material, the concept of forming limit strain in this paper refers to the necking strain. The experimental necking strains of a tube can be defined with different methods, such as the strain-rate method [9], the maximum pressure method [10], and the time-dependent method [11].

The forming limit diagram (FLD) is an important tool to evaluate the formability of thin-walled metals. The traditional strain-based FLD (*ε*-FLD) proposed by Keeler et al. [12] and Goodwin [13] is determined under linear loading paths. However, the *ε*-FLD has been shown to be path-dependent [14,15,16]. Thus, the *ε*-FLD obtained under linear loading paths are not suitable for the nonlinear loading conditions. In order to overcome the influence of the load path, several stress-based FLDs have been developed, for example, the forming limit stress diagram (FLSD) [17,18] and the extended stress FLD (XSFLD) [19]. Experimental results show that the path sensitivity of these FLDs is significantly reduced. However, due to the reduction of the slope of the true stress–strain curve as the strain increases, a small change in stress close to the necking limit may lead to a large change in strain. Moreover, the stress states may not be accurately measured directly. As a result, the application of these stress-based FLDs is limited.

Stoughton and Yoon [20] established the polar effective plastic strain FLD (PEPS-FLD) using the effective plastic strain to represent formability. The path in the PEPS-FLD is determined based on the magnitude of the effective plastic strain radius and the direction of the strain increment in the conventional strain diagram. Nguyen et al. [21] predicted necking in free-expansion hydroforming of the tube with tensile pre-straining in the axial direction and demonstrated that PEPS-FLD captured the forming limit accurately. The main benefits of the PEPS-FLD are (a) its weak strain path-dependency, (b) its similar shape to *ε*-FLD, and its ease of understanding [22]. However, experimental studies showed that the path dependence of PEPS-FLD depends on the specific material [23]. For some materials, the influence of pre-strains on the PEPS-FLD cannot be ignored. In this case, the PEPS-FLD determined under linear loading paths is not an appropriate criterion for limit strain prediction under complex loading conditions [23].

On the other hand, the theoretical prediction of the forming limit is also critical in the design and analysis of forming processes. At present, many theoretical models for forming limit prediction have been proposed. The M-K model is one of the most widely used models in the prediction of forming limit of thin-walled metals [24,25]. The model can be combined with any anisotropic yield criterion and hardening law [26,27] and is suitable for nonlinear loading conditions. In the traditional M-K model, the limit state is determined by the ratio of major strain increment in the groove to that in the uniform region. However, it is rather empirical and physically unsound to use a constant critical strain increment ratio as the failure criterion, considering that the major strain incremental ratio at failure may be influenced by the stress state [28].

In order to accurately predict the forming limit, the M-K model can be combined with a ductile fracture criterion (DFC), for example, the well-known Gurson model and Gurson–Tvergaard–Needleman model [29,30,31]. This type of M-K+DFC model has been discussed briefly in the reviews of Banabic et al. [25,32] and Zhang et al. [33]. However, the computational cost of a combination of the M-K model and a physical DFC is much more expensive than the traditional M-K model [34]. It is worth noting that a phenomenological DFC is generally a function of stress components and strain increments, which can predict forming limits with much lower computational costs and take the nonlinear loading path into account through incremental calculation [35,36,37]. Therefore, the combination of the M-K model and a phenomenological DFC is worth studying in the prediction of the forming limit under nonlinear loading paths.

For some metals, the fracture forming limit curve (FFLC) monotonically decreases from uniaxial tension to equibiaxial tension, which can be captured by classic DFCs such as the Clift criterion [38], the Cockcroft–Latham criterion [39], and the McClintock criterion [40]. However, the FFLCs of many materials may have more complex shapes, for example, a shape of “V” [41]. Lou et al. [42] proposed the DF2012 criterion, and both monotonically decreasing and V-shaped curves can be predicted by the DF2012 criterion. In our previous work, the FLCs predicted by different M-K+DFC models under linear loading conditions were compared, and the M-K+DF2012 model gave the best prediction [43]. However, the performance of an M-K+DFC model under nonlinear loading paths is also very important and has not been studied systematically. Therefore, it is necessary to carry out further studies on the combined model under nonlinear loading conditions.

In this work, an improved M-K+DF2012 model will be established first to predict the forming limit at the necking of an AA6061 aluminum alloy tube under nonlinear loading paths. Then, the limit strains of the tube under linear and nonlinear loading paths will be tested through tube-controllable biaxial loading experiments. After that, the validity of the M-K+DF2012 model will be validated by the experimental results. Finally, the effect of pre-strains on the *ε*-FLD and the PEPS-FLD predicted by the M-K+DF2012 model will be discussed.

## 2. M-K+DF2012 Model for Tubes

### 2.1. Fundamental Assumptions

**Figure 1:**
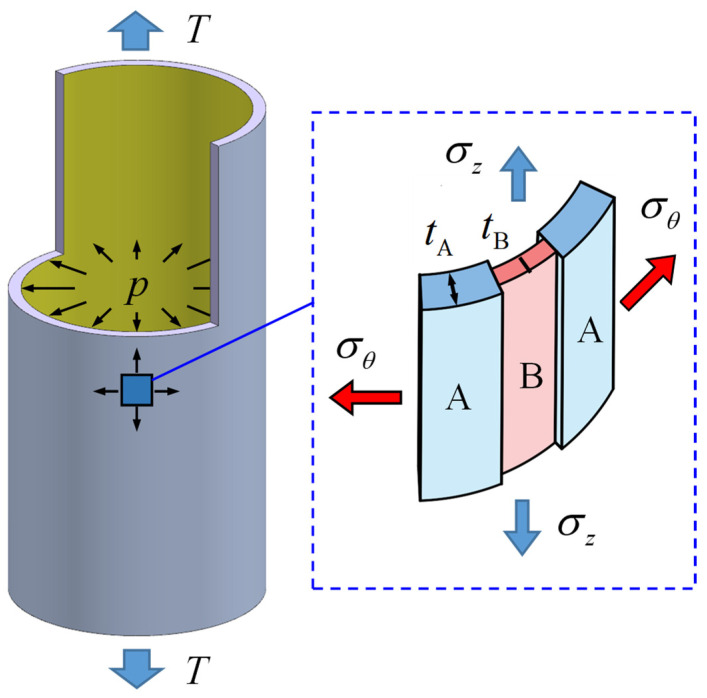
Schematic diagram of the axial groove in tubes.

In tube hydroforming, due to the action of internal pressure, the hoop strain is usually the largest principal strain and the crack is often formed along the axial direction. Meanwhile, the thickness distribution in the hoop direction of an extruded tube is not homogenized because of the eccentricity of the extrusion mandrel [44]. Therefore, a groove along the axial direction is assumed to represent the thickness inhomogeneity of the tube, as shown in Figure 1. The tube specimen is divided into a uniform region (region A) with thickness *t*_A_ and a groove (region B) with thickness *t*_B_. The initial coefficient of non-homogeneity *f*_0_ is defined as the ratio of the initial thicknesses of regions B and A:

(1) $$f_{0}=t_{B0}/t_{A0}$$

It is important to point out that the normal stress $\sigma_{n}$ is usually much smaller than the axial stress $\sigma_{z}$ and hoop stress $\sigma_{\theta }$ in the hydroforming of a thin-walled tube. Thus, the normal stress can be ignored, and the stress condition can be simplified to the plane stress state. At the same time, the force equilibrium condition along the hoop direction between regions A and B must be satisfied:

(2) $$\sigma_{\theta A}t_{A}=\sigma_{\theta B}t_{B}$$

where $\sigma_{\theta A}$ and $\sigma_{\theta B}$ are the hoop stresses in regions A and B, respectively.

On the other hand, the axial strain increments in regions A and B should be equal according to the requirement of coordinated deformation:

(3) $$d\varepsilon_{zA}=d\varepsilon_{zB}$$

In the traditional M-K model, when the ratio of the major strain increment becomes very large (for example, 10), the strains in region A are the limit strains. Differently, in the M-K+DF2012 model, the onset of fracture in region B is determined by the DF2012 criterion, and the strains in region A at the moment are defined as the limit strains, and the DF2012 criterion can be expressed as [42]:

(4) $$\int_{0}^{{\bar{\varepsilon }}_{f}}{\left(\frac{2\tau_{\max}}{\bar{\sigma }}\right)}^{C_{1}}{\left(\frac{\langle 1+3\eta \rangle }{2}\right)}^{C_{2}}\;d\bar{\varepsilon }=C_{3},\;\;\langle x\rangle =\left\{ \begin{array}{l} x\;\;\mathrm{when}\;x\geq 0\\ 0\;\;\mathrm{when}\;x<0 \end{array}\right.$$

where $\eta =\sigma_{m}/\bar{\sigma }$ is the stress triaxiality, $\bar{\sigma }$ is the equivalent stress, and $\sigma_{m}=\left(\sigma_{1}+\sigma_{2}+\sigma_{3}\right)/3$ is the hydrostatic stress; $\sigma_{1}$, $\sigma_{2}$ and $\sigma_{3}$ are the three principal stress components, and $\sigma_{1}>\sigma_{2}>\sigma_{3}$; $\tau_{\max}=\left(\sigma_{1}-\sigma_{3}\right)/2$ is the maximum shear stress; *C*_1_, *C*_2_, and *C*_3_ are material constants. For a thin-walled tube subjected to a combined load of internal pressure and axial tension, the typical stress states are $\sigma_{1}=\sigma_{\theta }$, $\sigma_{2}=\sigma_{z}$, and $\sigma_{3}=\sigma_{t}=0$.

Considering that both the M-K model and the DF2012 criterion can be used with anisotropic plastic constitutive models and nonlinear loading paths, the M-K+DF2012 model is also suitable for predicting the forming limits of anisotropic materials under nonlinear loading conditions.

### 2.2. Prediction Process

**Figure 2:**
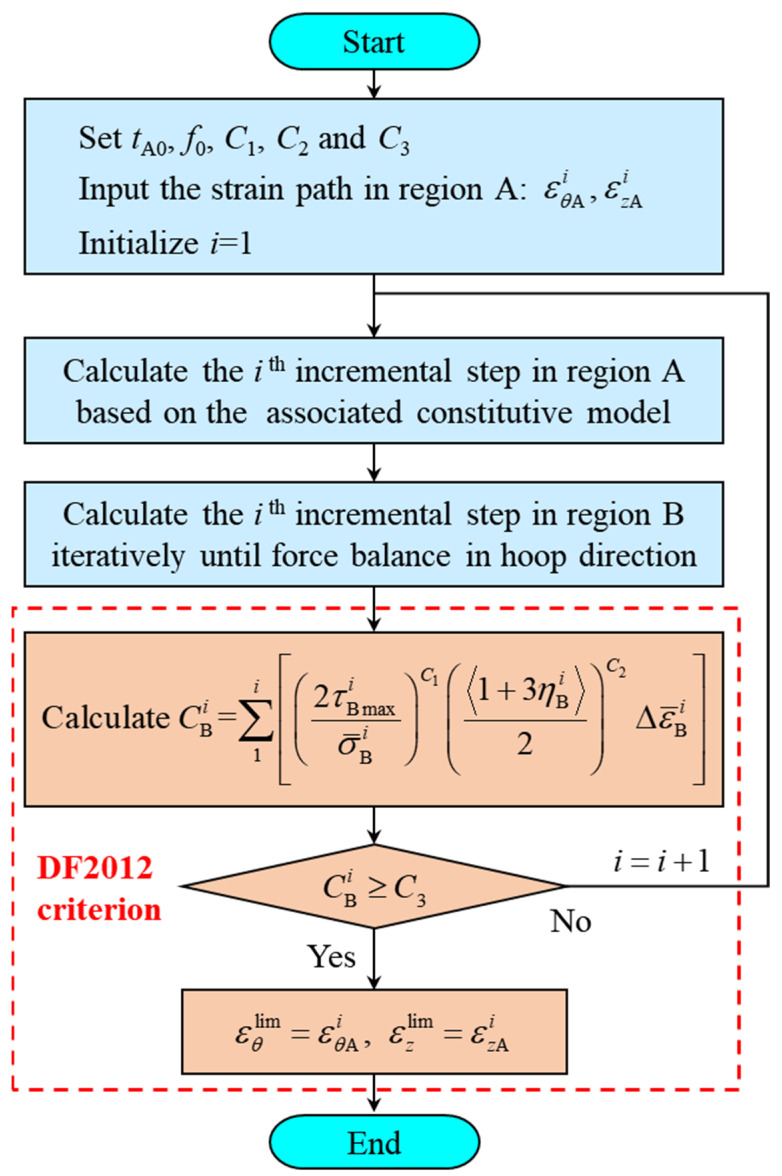
Prediction process for the limit strain under a nonlinear loading path using the M-K+DF2012 model.

The process of applying the M-K+DF2012 model to predict the limit strain under a nonlinear strain path is shown in Figure 2. Firstly, the strain path in region A should be discretized into sufficiently small incremental steps. According to the associated flow rule, the plastic potential equals the yield function ϕ, and there is a relationship between the strain increment ratio β and the stress state as follows:

(5) β=dεzdεθ=∂ϕ∂σz∂ϕ∂σθ

where $d\varepsilon_{\theta }$ and $d\varepsilon_{z}$ are the hoop and axial strain increments, respectively.

Hence, the stress components in region A at step *i* can be calculated based on the strain increments and the associated constitutive model. Then, the strain increments and stress components in region B at step *i* can be calculated as follows: The axial strain increment in region B, $d\varepsilon_{zB}$, equals that in region A; and the hoop strain increment $d\varepsilon_{\theta B}$, the axial stress $\sigma_{zB}$, and the hoop stress $\sigma_{\theta B}$ can be obtained by numerical iteration using force balance in the hoop direction, as seen in Equation (2). In the meantime, the equivalent strain increment $d\bar{\varepsilon }$ can be obtained based on the rule of the equal plastic work rate [45]:

(6) $$dW=\sigma_{\theta }d\varepsilon_{\theta }+\sigma_{z}d\varepsilon_{z}=\bar{\sigma }\left(\bar{\varepsilon }\right)d\bar{\varepsilon }$$

Therefore, the stress and strain components in regions A and B can be calculated step by step through the incremental method until the deformation in region B satisfies the DF2012 criterion. The strain in region A at that moment is the limit strain at necking under the given loading path.

### 2.3. Constitutive Model

A reasonable constitutive model is critical for the prediction of the forming limits. Here, an associated constitutive model with the Yld2000-2d anisotropic yield criterion and the power hardening law is adopted. The Yld2000-2d criterion is an advanced and commonly used yield criterion for anisotropic aluminum alloys [46], which is defined as:

(7) $$\phi ={\left|X_{1}^{\prime }-X_{2}^{\prime }\right|}^{k}+{\left|X_{1}^{\prime \prime }+2X_{2}^{\prime \prime }\right|}^{k}+{\left|2X_{1}^{\prime \prime }+X_{2}^{\prime \prime }\right|}^{k}=2{\bar{\sigma }}^{k}$$

where $X_{i}^{\prime }$ and $X_{i}^{\prime \prime }$ (*i* = 1, 2) are the principal values of tensors $X^{\prime }=L^{\prime }\cdot \sigma$ and $X^{\prime \prime }=L^{\prime \prime }\cdot \sigma$, respectively. σ, $L^{\prime }$, and $L^{\prime \prime }$ can be written as:

(8) $$\left[\sigma \right]={\left[\begin{array}{ccc} \sigma_{\theta } & \sigma_{z} & \sigma_{\theta z} \end{array}\right]}^{T}$$

(9) $$\left[L^{\prime }\right]=\left[\begin{array}{ccc} L_{11}^{\prime } & L_{12}^{\prime } & 0\\ L_{21}^{\prime } & L_{22}^{\prime } & 0\\ 0 & 0 & L_{66}^{\prime } \end{array}\right]=\frac{1}{3}\left[\begin{array}{ccc} 2\alpha_{1} & -\alpha_{1} & 0\\ -\alpha_{2} & 2\alpha_{2} & 0\\ 0 & 0 & \alpha_{7} \end{array}\right]$$

(10) $$\left[L^{\prime \prime }\right]=\left[\begin{array}{ccc} L_{11}^{\prime \prime } & L_{12}^{\prime \prime } & 0\\ L_{21}^{\prime \prime } & L_{22}^{\prime \prime } & 0\\ 0 & 0 & L_{66}^{\prime \prime } \end{array}\right]=\frac{1}{9}\left[\begin{array}{ccc} 8\alpha_{5}-2\alpha_{3}-2\alpha_{6}+2\alpha_{4} & 4\alpha_{6}-4\alpha_{4}-4\alpha_{5}+\alpha_{3} & 0\\ 4\alpha_{3}-4\alpha_{5}-4\alpha_{4}+\alpha_{6} & 8\alpha_{4}-2\alpha_{6}-2\alpha_{3}+2\alpha_{5} & 0\\ 0 & 0 & 9\alpha_{8} \end{array}\right]$$

in which $\alpha_{i}$ (*i* = 1, 2…8) represents pending coefficients.

The principal values of **X** are

(11) $$\left\{ \begin{array}{l} X_{1}=\frac{1}{2}\left(X_{11}+X_{22}+\sqrt{{\left(X_{11}-X_{22}\right)}^{2}+4X_{12}^{2}}\right)\\ X_{2}=\frac{1}{2}\left(X_{11}+X_{22}-\sqrt{{\left(X_{11}-X_{22}\right)}^{2}+4X_{12}^{2}}\right) \end{array}\right.$$

On the other hand, the widely used power-hardening law is as follows:

(12) $$\bar{\sigma }=K{\bar{\varepsilon }}^{n}$$

where *K* and *n* are the hardening coefficient and strain-hardening exponent, respectively.

## 3. Experiments

### 3.1. Experimental Principle and Setup

**Figure 3:**
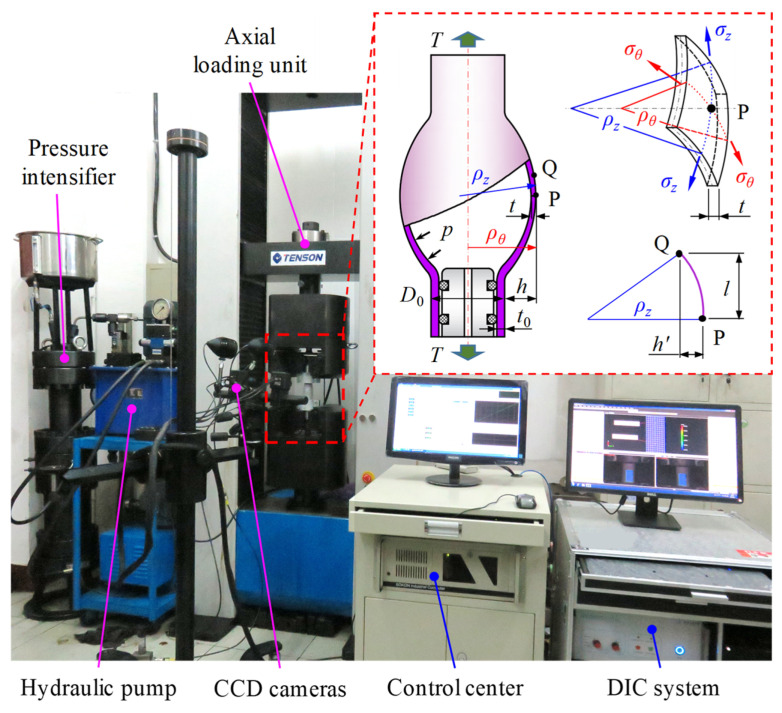
Principle and setup of tube controllable biaxial loading test.

In this paper, the limit strains of the tube were tested in tube-controllable biaxial loading experiments, of which the principle and setup are shown in Figure 3. The tube specimen is bulged by the combined load of internal pressure *p* and axial load *T*. The deformation process of the specimen can be measured and recorded by the digital image correlation (DIC) system in real time, and the two principal stresses $\sigma_{z}$ and $\sigma_{\theta }$ at the central point of the specimen can be controlled in real time by controlling *p* and *T*.

The stress state of the tube can be analyzed with the aid of the membrane theory if the ratio of thickness to diameter is less than 1/20 [47]. The axial stress $\sigma_{z}$ and hoop stress $\sigma_{\theta }$ can be calculated by the following equations [48].

(13) $$\left\{ \begin{array}{l} \sigma_{z}=\frac{p\pi {\left(\rho_{\theta }-t\right)}^{2}+T}{\pi \left(2\rho_{\theta }-t\right)t}\\ \sigma_{\theta }=\frac{2(\rho_{z}-t)(\rho_{\theta }-t)}{(2\rho_{z}-t)t}p-\frac{2\rho_{\theta }-t}{2\rho_{z}-t}\sigma_{z} \end{array}\right.$$

where *t*, $\rho_{\theta }$ and $\rho_{z}$ are the instantaneous thickness, hoop, and axial radii of curvature at point P, respectively.

According to the geometric relationship, the values of $\rho_{\theta }$ and $\rho_{z}$ can be calculated by Equations (14) and (15), respectively.

(14) $$\rho_{\theta }=\frac{D_{0}}{2}+h$$

(15) $$\rho_{z}=\frac{{h^{\prime }}^{2}+l^{2}}{2h^{\prime }}$$

where *D*_0_ is the initial diameter of the tube, *h* is the bulging height of point P, *l* and $h^{\prime }$ are the axial distance and the radial distance between points P and Q, and the axial profile of the tube specimen is assumed to be circular, as can be seen in Figure 3.

In addition, the instantaneous thickness *t* at point P can be calculated by

(16) $$t=t_{0}\exp\left(-\varepsilon_{z}-\varepsilon_{\theta }\right)$$

where *t*_0_ is the initial thickness of the tube.

The setup shown in Figure 3 consists of four units: Axial loading, internal pressure, displacement and strain measurement, and the control center. The axial loading unit acting on the ends of the tube is an electronic universal testing machine with a load capacity of ±200 kN. The internal pressure unit is a pressure intensifier with a maximum pressure of 40 MPa, which provides the internal pressure required for bulging. The displacement and strain measurement unit is a three-dimensional DIC system developed by the Institute of Mould and Advanced Forming Technology of Xi’an Jiaotong University. The control center unit is established using an industrial computer to achieve the simultaneous functioning of the above three units.

**Figure 4:**
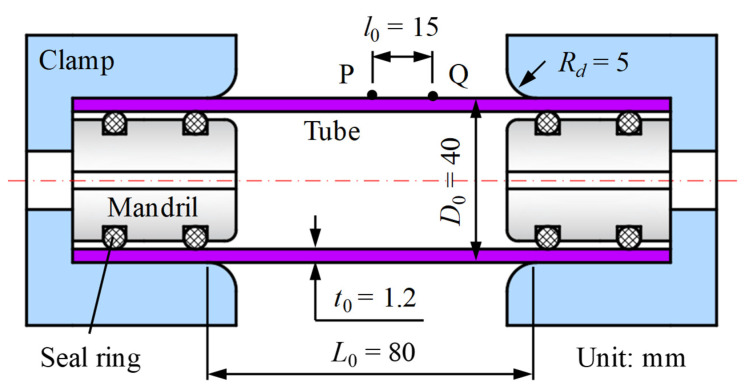
Schematic diagram of the tube specimen.

In this work, an extruded aluminum alloy seamless tube of AA6061 with a diameter of 40 mm and a thickness of 1.2 mm was used. As shown in Figure 4, the initial gauge length *L*_0_ of the tube specimen was designed to be 80 mm, which was twice the initial outer diameter of the tube. Before testing, a speckle pattern was sprayed on the surface of the specimen to record the deformation process by DIC technology. In the meantime, the central point P and another point Q were marked with an initial axial distance of *l*_0_ = 15 mm.

### 3.2. Scheme of Loading Paths

In this paper, the *ε*-FLD of the tube under linear loading conditions was tested first because it serves as the reference for discussing the influence of loading paths on the limit strains. In order to obtain the forming limit strains from hoop uniaxial tension to equibiaxial tension, nine linear loading tests were carried out with the axial–hoop stress ratios $\sigma_{z}/\sigma_{\theta }$ = 0, 0.125, 0.25, 0.375, 0.5, 0.625, 0.75, 0.875, and 1.0.

**Figure 5:**
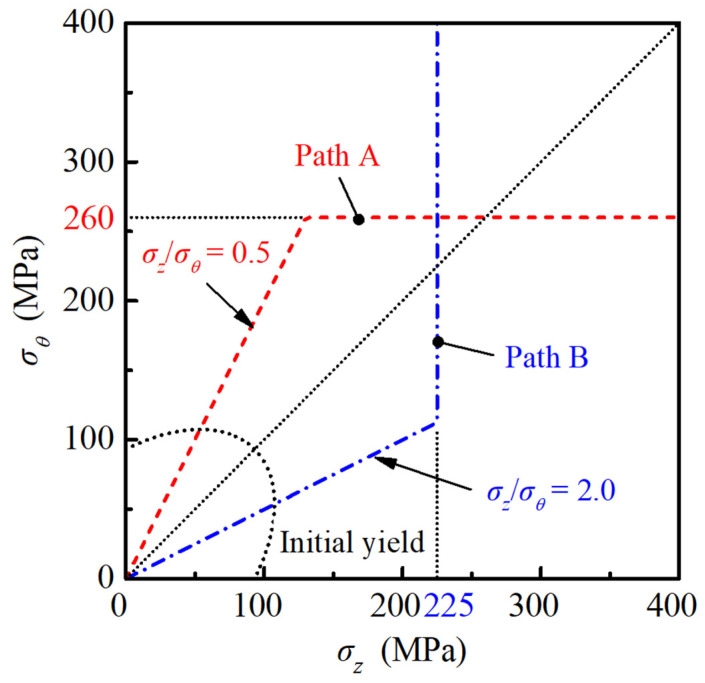
Designed continuous nonlinear loading paths for the AA6061 tube.

Pre-bulging and bending are two important pre-deformation forms in tube hydroforming, of which the strain states are approximately hoop plane strain and axial plane strain, respectively. In order to analyze the influence of plane strain pre-deformation and verify the validity of the M-K+DF2012 model under nonlinear loading paths, two nonlinear stress loading paths with plane strain pre-deformation were designed, as shown in Figure 5. In the first stage of path A, the stress ratio is prescribed as $\sigma_{z}/\sigma_{\theta }$ = 0.5, while the hoop stress remains constant in the second stage, and the turning point is located at (130, 260) MPa. For path B, the stress state in the first stage is $\sigma_{z}/\sigma_{\theta }$ = 2.0, the axial stress remains constant in the second stage, and the turning point is located at (225, 112.5) MPa. The stress states $\sigma_{z}/\sigma_{\theta }$ = 0.5 and 2.0 correspond to the hoop plane strain tension and axial plane strain tension for an isotropic tube, respectively.

## 4. Results

### 4.1. Material Properties

Three tests were carried out to test the mechanical properties of the AA6061 tube, i.e., an axial uniaxial tensile test on a strip-shape specimen cut from the tube (refer to ASTM: E8), a controllable biaxial loading test with $\sigma_{z}/\sigma_{\theta }=0$ as the alternative to the hoop uniaxial tension, and an equibiaxial tensile test with $\sigma_{z}/\sigma_{\theta }=1.0$. Anisotropic parameters of the tube deduced from the experimental results are listed in Table 1. $r_{\;b}=\varepsilon_{z}/\varepsilon_{\theta }$ is the biaxial anisotropy coefficient under the loading condition of $\sigma_{z}/\sigma_{\theta }=1.0$. Here, $\varepsilon_{\theta }$ and $\sigma_{\theta 0}$ were chosen as the denominators considering that the major limit strain is usually in the hoop direction.

**Figure 6:**
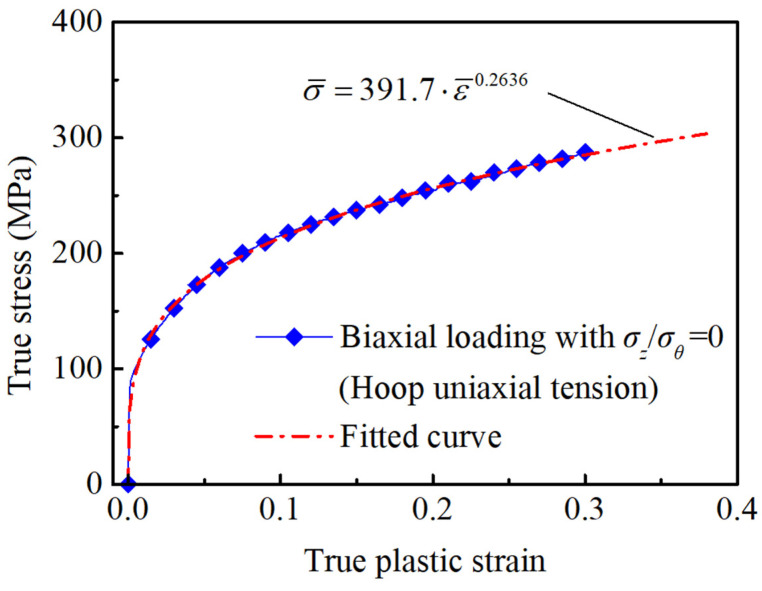
Stress–strain curve of the AA6061 tube in hoop direction.

Meanwhile, the power hardening law of the tube was obtained by fitting the hoop true stress–plastic strain curve, the obtained values of *K* and *n* are also given in Table 1. As shown in Figure 6, the fitted hardening curve is in good agreement with the experimental stress–strain curve of the tube. The coefficients *α*_1_ to *α*_6_ of the Yld2000-2d yield criterion for the AA6061 tube are determined iteratively according to the *r*-values and stress ratios in Table 1. During the calculation, the exponent *k* was set to 8 considering that the aluminum alloy is an FCC material, and the coefficients *α*_7_ and *α*_8_ were set to 1.0 because the two coefficients are related to the shear stress components and do not influence the prediction of the tube controllable biaxial loading process [49]. The obtained values of *α*_1_ to *α*_6_ are also listed in Table 1.

### 4.2. Experimental Limit Strains

**Figure 7:**
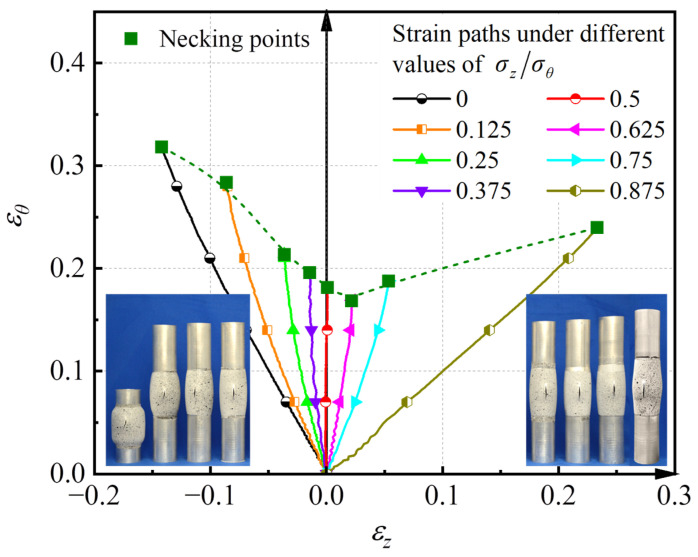
Necking points and strain paths of the AA6061 tube under linear loading paths.

According to the time-dependent method proposed by Martínez-Donaire et al. [11], the onset of necking corresponds to the occurrence of a maximum of the first derivative of $\varepsilon_{\theta }$ at the boundary of the instability region. The limit strains at necking and corresponding strain paths at the central point of tube specimens under loading conditions of $\sigma_{z}/\sigma_{\theta }$ = 0, 0.125, 0.25, 0.375, 0.50, 0.625, 0.75, and 0.875 are shown in Figure 7. The necking point under the condition of $\sigma_{z}/\sigma_{\theta }$ = 1.0 is not given because the crack is along the hoop direction, which means that the assumption of the groove along the axial direction is not satisfied for this case. It can be seen from Figure 7 that these strain paths are all approximately linear and the experimental forming limit curve (FLC) is V-shaped.

**Figure 8:**
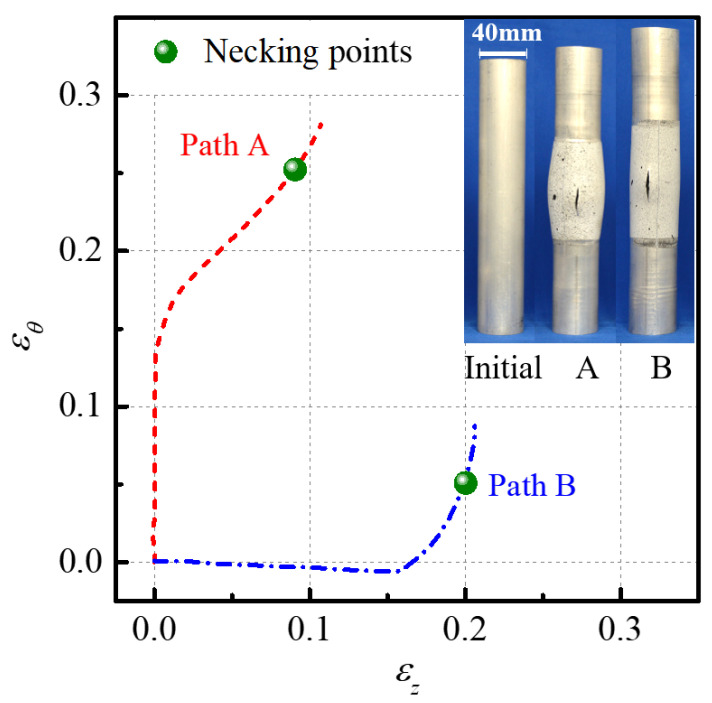
Necking points and strain paths of the AA6061 tube under nonlinear loading paths.

Figure 8 shows the experimental limit strain points and corresponding strain paths of the AA6061 tube under nonlinear loading paths A and B. Both the two strain paths in the first stage are linear and close to the state of plane strain. While in the second stage, the strain paths gradually deviate from plane strain. Finally, both nonlinear strain paths converge to the plane strain state of $d\varepsilon_{z}=0$ after necking, which leads to the axial cracks in the two specimens. It can be also noted that the limit strain point under the nonlinear loading path A is obviously higher than the experimental FLC in Figure 7 under linear loading conditions, but the limit strain point under path B is lower. It indicates that the formability of the AA6061 tube is significantly affected by the loading path.

### 4.3. Prediction of Limit Strains

In order to predict the forming limit of the AA6061 tube using the M-K+DF2012 model, material constants *C*_1_, *C*_2_, and *C*_3_ in the DF2012 criterion and the initial coefficient of non-homogeneity *f*_0_ of the tube must be determined first. The values of these four parameters are listed in Table 2, which were determined by an optimization method aiming at the minimum root mean squared error between the experimental hoop limit strains shown in Figure 7 and the corresponding predicted values. The experimental data under nonlinear loading paths in Figure 8 are not included in the optimization.

**Figure 9:**
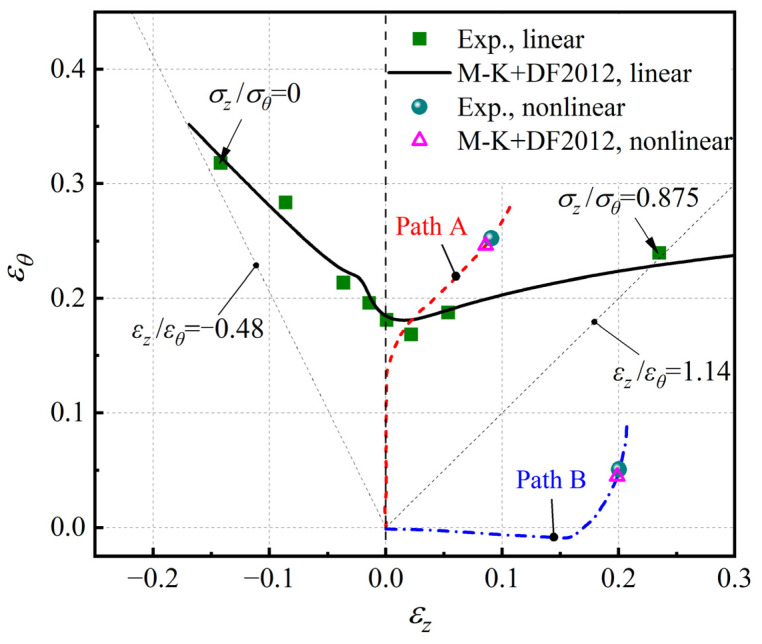
Comparison of the predicted and experimental limit strains of the AA6061 tube.

In Figure 9, the limit strains of the tube predicted by the M-K+DF2012 model are compared with the experimental strain points. The M-K+DF2012 model provides accurate predictions under all eight linear loading paths and the two nonlinear loading paths. It indicates that the M-K-DF2012 model determined by only the data of linear loading can reasonably predict the influence of the nonlinear loading path on the forming limit of the AA6061 tube.

## 5. Discussion

### 5.1. Effect of Pre-Strain on Predicted ε-FLD

In tube hydroforming, proper pre-deformation steps are usually required, and the strain state of the pre-deformation is primarily selected in the range of hoop uniaxial pre-straining to axial plane strain pre-straining. In order to analyze the influence of pre-strains on the limit strains, the *ε*-FLCs of the AA6061 tube under four different pre-straining modes, namely, hoop uniaxial tensile pre-straining (UT-*θ*), hoop plane strain tensile pre-straining (PT-*θ*), biaxial tensile pre-straining with $\varepsilon_{z}=\varepsilon_{\theta }$ (BT), and axial plane strain tensile pre-straining (PT-*z*), were predicted using the M-K+DF2012 model.

The nonlinear loading path is the two subsequent linear loading steps described by the following relation:

(17) $$\left\{ \begin{array}{l} \beta =\frac{d\varepsilon_{z}}{d\varepsilon_{\theta }}=\beta_{1}\;,\;\;\mathrm{If}\;\;\bar{\varepsilon }\leq {\bar{\varepsilon }}^{*}\\ \beta =\frac{d\varepsilon_{z}}{d\varepsilon_{\theta }}=\beta_{2}\;,\;\;\mathrm{If}\;\;\bar{\varepsilon }>{\bar{\varepsilon }}^{*} \end{array}\right.$$

where $\beta_{1}$ and $\beta_{2}$ are the strain increment ratios related to the pre-strain step and the second loading step, respectively, and ${\bar{\varepsilon }}^{*}$ is the equivalent strain of the pre-deformation in region A.

**Figure 10:**
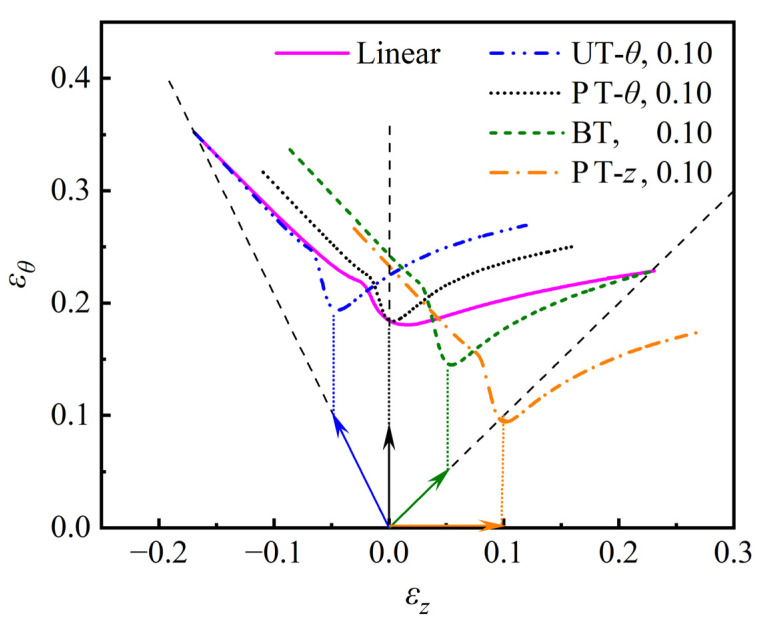
*ε*-FLCs of the AA6061 tube predicted by the M-K+DF2012 model under different pre-strain conditions.

Figure 10 shows *ε*-FLCs of the tube predicted by the M-K+DF2012 model under different pre-strain conditions, where the equivalent pre-strains are all set to ${\bar{\varepsilon }}^{*}$ = 0.1. It can be easily found that *ε*-FLCs are obviously affected by pre-strains. Compared with the *ε*-FLC under linear loading conditions, the *ε*-FLC after UT-*θ* pre-straining shifts towards the tension-compression side. The *ε*-FLC after PT-*θ* pre-straining increases slightly in the tension-compression side and increases obviously in the tension-tension side but remains unchanged in the hoop plane strain tensile state. Meanwhile, the *ε*-FLCs after BT pre-straining and PT-*z* pre-straining shift towards the tension-tension side with a significant reduction of hoop strain. The moving direction of the *ε*-FLC predicted under different pre-strains is approximately consistent with the experimental results in the literature [23].

### 5.2. Effect of Pre-Strain on Predicted PEPS-FLD

**Figure 11:**
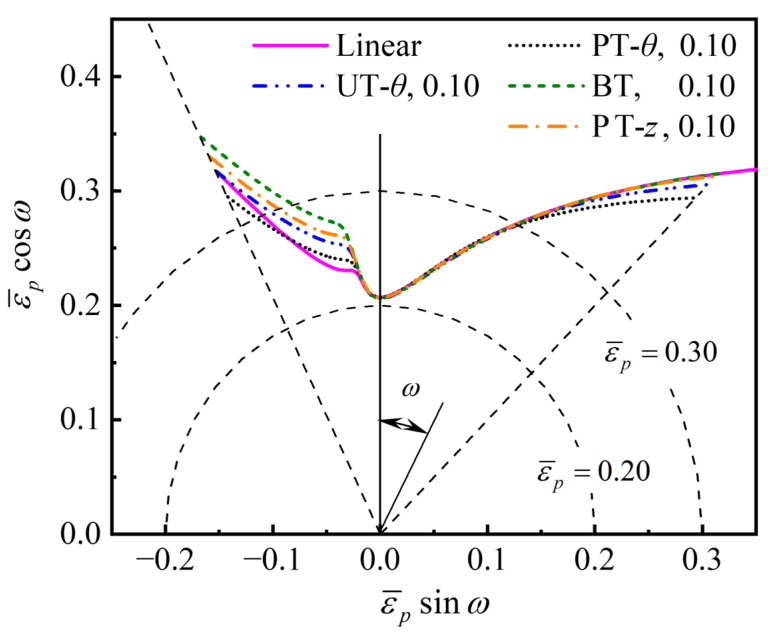
Predicted PEPS-FLD of the AA6061 tube under different pre-strain conditions.

In order to investigate the effect of the loading path on the PEPS-FLD predicted by the M-K+DF2012 model, the *ε*-FLCs in Figure 10 were converted to PEPS-FLCs as shown in Figure 11. It was observed that the PEPS-FLD is weakly strain path-dependent. On the right-hand side of the FLD, all PEPS-FLCs almost fall on a single curve. On the left-hand side of the FLD, the equivalent limit strain at necking increases slightly after pre-straining such as UT-*θ*, BT, and PT-*z*, while the effect of the PT-*θ* pre-straining on the equivalent limit strain is small. Therefore, if the final deformation state of the AA6061 tube is located on the right-hand side of the PEPS-FLD, e.g., a tension–tension strain state, the forming limit can be evaluated through the PEPS-FLD regardless of the loading paths.

**Figure 12:**
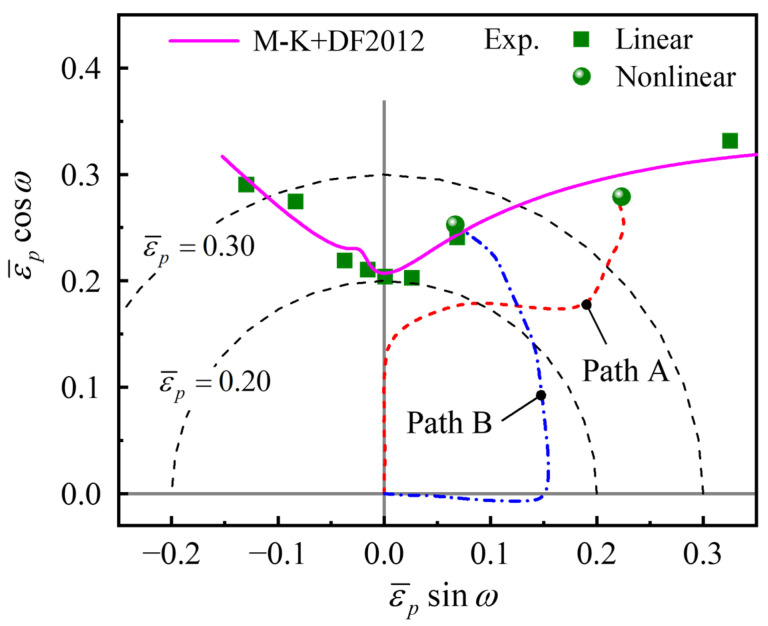
Comparison of predicted PEPS-FLC and experimental limit strains of the AA6061 tube.

Then, the PEPS-FLC predicted by the M-K+DF2012 model under linear loading conditions is compared with the experimental results under linear paths and nonlinear paths A and B, as shown in Figure 12. The two experimental points of nonlinear loading are on the right-hand side, and all the points on the right-hand side roughly follow the same curve. It indicates that the path dependence of the PEPS-FLD of the AA6061 tube on the right-hand side is weak. In other words, the small difference between the predicted curves on the right-hand side of Figure 11 is consistent with the experimental phenomenon.

## 6. Conclusions

In this paper, the use of the M-K+DF2012 model for the prediction of forming limits under nonlinear loading paths was established, the limit strains of the AA6061 aluminum alloy tube under a set of linear and two typical nonlinear loading paths were tested, the validity of the M-K+DF2012 model was validated by the experimental results, and the *ε*-FLD and PEPS-FLD under different pre-strains were discussed. The following conclusions were obtained:

(1) The forming limit of the AA6061 tube is significantly affected by the loading path. The limit strain in the tension–tension strain state increases obviously after a hoop plane strain tensile pre-straining.

(2) The M-K+DF2012 model can reasonably predict the limit strains of the AA6061 aluminum alloy tube under both linear and nonlinear loading paths.

(3) The *ε*-FLD predicted by the M-K+DF2012 model under different pre-strain conditions shows a strong dependence on the pre-strain, and the direction in which the FLC shifts relative to the conventional FLC under linear loading conditions depends on the types of pre-strain.

(4) The PEPS-FLD predicted by the M-K+DF2012 model is weakly strain path dependent, and almost path-independent on the right-hand side for the AA6061 tube. The curve predicted under linear loading conditions is in good agreement with the experimental limit strain points of the AA6061 tube.

In the future, it is worth applying the M-K+DF2012 model to different materials such as Magnesium alloy and Titanium alloy and improving the model predictability according to the characteristics of the materials. On the other hand, more influential factors should be introduced into the model in order to predict the forming limit under more complex deformation conditions. The influential factor could be the angle of the groove, the strain rate sensitivity, and the stress in the thickness direction.

## Figures and Tables

**Table 1:** Mechanical properties and the Yld2000-2d yield criterion of the AA6061 tube (Adapted from [43]).

Parameters	Anisotropic Parameters					Power Hardening		Coefficients of the Yld2000-2d Yield Criterion					
	$r_{z}$	$r_{\theta }$	$r_{\;b}$	$\sigma_{z0}/\sigma_{\theta 0}$	$\sigma_{b0}/\sigma_{\theta 0}$	*K* (MPa)	*n*	$\alpha_{1}$	$\alpha_{2}$	$\alpha_{3}$	$\alpha_{4}$	$\alpha_{5}$	$\alpha_{6}$
Values	0.454	0.927	2.80	0.988	0.959	391.7	0.2636	1.0072	0.9436	1.1625	1.0764	0.9725	0.8519

**Table 2:** Parameter values of the M-K+DF2012 model for the AA6061 tube.

Parameters	*C*_1_	*C*_2_	*C*_3_	*f*_0_
Values	10.77	−2.814	0.4114	0.9852

## Data Availability

Not applicable.
